# Complete Genome Sequence of a Porcine Epidemic Diarrhea Virus Isolated in Belgorod, Russia, in 2008

**DOI:** 10.1128/genomeA.01026-17

**Published:** 2017-10-12

**Authors:** Olga Strizhakova, Dennis Hanke, Ilya Titov, Sandra Blome, Alexander Malogolovkin

**Affiliations:** a Federal Research Center for Virology and Microbiology (FRCVM), Pokrov, Russia; b Federal Research Institute for Animal Health, the Friedrich-Loeffler-Institut (FLI), Greifswald-Insel Riems, Germany

## Abstract

We identified porcine epidemic diarrhea virus (PEDV) in stool samples from sick piglets in the Belgorod region of Russia. The complete coding genome sequence of 28,295 nucleotides (nt) of PEDV was generated. Compared to a prototype PEDV strain (DR13), an extreme number of mismatches in the S gene were revealed.

## GENOME ANNOUNCEMENT

Porcine epidemic diarrhea virus (PEDV) belongs to the genus *Alphacoronavirus* within the *Coronaviridae* virus family. PEDV caused a vast number of outbreaks and tremendous losses in the United States in 2013 to 2014 (1, 2). Porcine epidemic diarrhea (PED) was also registered in Europe and Russia. The European PEDV strains are closely related to each other and form a homogeneous S-INDEL cluster. Nevertheless, a minor genetic shift in terms of virus evolution has been shown recently (3).

In Russia, several PEDV isolates were identified between 2005 and 2008 and partially sequenced (4). However, there are no data of complete genome sequences of Russian PEDV isolates publicly available to date.

Here, we had the opportunity to investigate a PEDV isolate (PEDV/Belgorod/dom/2008) from the Belgorod region that caused a severe outbreak in 1-month old piglets in 2008.

Next-generation sequencing was done with an Illumina MiSeq instrument with MiSeq reagent kit v3 in 2- × 300-bp PE mode (Illumina, San Diego, CA, USA). Sequence assembly, the subsequent mapping of the raw sequence data, and the analysis of the resulting sequences were done with the Genome Sequencer software suite (v3.0; Roche) and the Geneious software suite (v8.1.3; Biomatters Ltd., Auckland, New Zealand) as previously described (3). The resulting sequence, PEDV/Belgorod/dom/2008, was annotated using the RAST server (5).

The complete coding sequence of the isolate is 28,295 nucleotides (nt) in length including a poly(A) tail. The genomic organization of the isolate is similar to the canonical PEDV structure described elsewhere (6, 7). The most similar PEDV sequences at the whole-genome level were among Chinese PEDV isolates (i.e., KX791060, KC210147, KC210145, and KT021229) with 97% identity and an E value of 0.0. However, the spike protein sequence (S gene) (nt 20,633 to 24,751) of PEDV/Belgorod/dom/2008 had no matches using a megaBLAST search. Only 66% identity with other Asian PEDV strains (i.e., KJ857459, KJ657476, KJ451036, and KC764954) was revealed using a BLASTn search. In addition, the nucleoprotein sequence (N gene) (26,336 to 27,640) of the isolate had a 21-nt deletion (26,978 to 26,999) compared to prototype DR13 PEDV strains.

Overall, PEDV/Belgorod/dom/2008 has high nucleotide identity (97%) at the whole-genome level with other PEDV strains circulating in Asia, but has a unique spike protein sequence which has low similarity (66%) with PEDV sequences available in GenBank.

The genome sequence reported here will help us to understand the evolutionary characteristics and molecular epidemiology of PEDV in different parts of the world.

### Accession number(s).

The complete PEDV/Belgorod/dom/2008 genome sequence has been deposited in GenBank under accession number MF577027.

## References

[1] Stevenson GW, Hoang H, Schwartz KJ, Burrough ER, Sun D, Madson D, Cooper VL, Pillatzki A, Gauger P, Schmitt BJ, Koster LG, Killian ML, Yoon KJ 2013 Emergence of porcine epidemic diarrhea virus in the United States: clinical signs, lesions, and viral genomic sequences. J Vet Diagn Invest 25:649–654. doi:10.1177/1040638713501675. 23963154

[2] Huang YW, Dickerman AW, Piñeyro P, Li L, Fang L, Kiehne R, Opriessnig T, Meng XJ 2013 Origin, evolution, and genotyping of emergent porcine epidemic diarrhea virus strains in the United States. mBio 4:e00737-13. doi:10.1128/mBio.00737-13. 24129257 PMC3812708

[3] Hanke D, Pohlmann A, Sauter-Louis C, Höper D, Stadler J, Ritzmann M, Steinrigl A, Schwarz BA, Akimkin V, Fux R, Blome S, Beer M 2017 Porcine epidemic diarrhea in Europe: in-detail analyses of disease dynamics and molecular epidemiology. Viruses 9:177. doi:10.3390/v9070177. PMC5537669 28684708

[4] Ben Salem AN, Chupin Sergei A, Bjadovskaya Olga P, Andreeva Olga G, Mahjoub A, Prokhvatilova Larissa B 2010 Multiplex nested RT-PCR for the detection of porcine enteric viruses. J Virol Methods 165:283–293. doi:10.1016/j.jviromet.2010.02.010. 20170679 PMC7112813

[5] Aziz RK, Bartels D, Best AA, DeJongh M, Disz T, Edwards RA, Formsma K, Gerdes S, Glass EM, Kubal M, Meyer F, Olsen GJ, Olson R, Osterman AL, Overbeek RA, McNeil LK, Paarmann D, Paczian T, Parrello B, Pusch GD, Reich C, Stevens R, Vassieva O, Vonstein V, Wilke A, Zagnitko O 2008 The RAST Server: Rapid Annotations using Subsystems Technology. BMC Genomics 9:75. doi:10.1186/1471-2164-9-75. 18261238 PMC2265698

[6] Qin Y, Lu B, He Y, Li B, Duan Q, Liang J, Chen Z, Su Q, Bi B, Zhao W 2017 Full-length genome sequence of porcine epidemic diarrhea virus strain CH/GX/2015/750A. Genome Announc 5(27):e00361-17. doi:10.1128/genomeA.00361-17. 28684562 PMC5502843

[7] Lee S, Lee C 2017 Complete genome sequence of a novel S-insertion variant of porcine epidemic diarrhea virus from South Korea. Arch Virol 162:2919–2922. doi:10.1007/s00705-017-3441-y. PMC7086619 28589511

